# Achieving reductions in the unmet need for contraception with postpartum family planning counselling in Ethiopia, 2019–2020: a national longitudinal study

**DOI:** 10.1186/s13690-023-01096-1

**Published:** 2023-05-01

**Authors:** Kalayu Brhane Mruts, Gizachew A Tessema, Nigussie Assefa Kassaw, Amanuel Tesfay Gebremedhin, Jane A Scott, Gavin Pereira

**Affiliations:** 1 School of Public Health, Debre Berhan University, P.O. Box 445, Debre Berhan, Ethiopia; 2 Curtin School of Population Health, Curtin University, Perth, Australia; 3 School of Public Health, University of Adelaide, Adelaide, Australia; 4 School of Public Health, College of Health Sciences, Addis Ababa University, Addis Ababa, Ethiopia; 5 Centre for Fertility and Health (CeFH), Norwegian Institute of Public Health, Oslo, Norway; 6 enAble Institute, Curtin University, Perth, Australia

**Keywords:** Ethiopia, Unmet need, Family planning counselling, Postpartum

## Abstract

**Background:**

An unmet need for contraception is associated with unintended pregnancy and adverse maternal and childhood outcomes. Family planning counselling is linked with reduced unmet need for contraception. However, evidence is lacking in Ethiopia on the impact of integrated family planning counselling on the unmet need for contraception. This study aimed to examine the association between family planning counselling and the unmet need for contraception in Ethiopia.

**Methods:**

We used community-based prospective cohort study data from a nationally representative survey conducted by Performance Monitoring for Action Ethiopia between 2019 and 2020. Women who had received three maternal and child health (MCH) services (n = 769) - antenatal care (ANC), facility delivery and child immunisation - were included in this study. The primary exposure variable was family planning counselling provided during the different MCH services. A weighted modified Poisson regression model was used to estimate the adjusted relative risk (aRR) of the unmet need for contraception.

**Results:**

The prevalence of family planning counselling during ANC, prior to discharge, and child immunisation was 22%, 28%, and 28%, respectively. Approximately one-third (34%) of the women had an unmet need for contraception. Family planning counselling prior to discharge from the facility was associated with reductions in the unmet need for contraception (aRR 0.88; 95% CI 0.67, 1.16). The risk of unmet need for contraception was 31% (aRR 0.69; 95% CI 0.48, 0.98) less likely among women who had received family planning counselling during child immunisation services. However, family planning counselling during ANC was associated with an increased unmet need for contraception (aRR 1.24; 95% CI 0.93, 1.64).

**Conclusion:**

Strongest evidence was observed for moderate associations between reductions in the unmet need for contraception and family planning counselling during the provision of child immunisation services in Ethiopia.

**Supplementary Information:**

The online version contains supplementary material available at 10.1186/s13690-023-01096-1.

## Background

An unmet need for contraception is linked with unintended pregnancy and adverse consequences on mothers and birth outcomes [1, 2]. Conceptually, unmet need is defined as not wanting to become pregnant but not using contraceptive methods while interested in using [3]. Practically, the retrospective approach, used by the Demographic and Health Survey (DHS), has been used for estimating the unmet need for contraception for a long time [4, 5]. However, as this approach estimates unmet needs based on the intendedness of the last birth, it does not reflect the current risk of unintended pregnancy [5]. Due to this reason, the retrospective approach underestimates the unmet need for contraception among women in the postpartum period [6]. The current status approach is another alternative used to estimate an unmet need for contraception among women in the postpartum period, which assumes women as having an unmet need for contraception if they resume sexual intercourse and menstrual cycle following their recent birth and do not want subsequent pregnancy within 24 months, but are not using any contraceptive methods [7]. Evidence indicated that this approach demonstrates conservative estimates of the unmet need for contraception [6, 7].

The unmet need for contraception is one of family planning programs’ core performance and advocacy indicators [8]. Globally, the unmet need for contraception has declined in the past decades [9], although still, 257 million women have an unmet need for modern contraception [10], of which three-fourths of them are from low-and middle-income countries (LMICs) [2]. The unmet need for contraception is linked with unintended pregnancy [1, 11], which in turn leads to adverse perinatal outcomes, including abortion, stillbirth, preterm birth, low birth weight, and neonatal mortality [2]. About half (49%) of pregnancies in LMICs are unintended [2]. A study conducted on 36 LMIC’s using DHS data indicated that above 65 per cent of women with unintended pregnancies were either non-contraceptive user or traditional method users [12]. The unmet need for contraception also contributes to high fertility and population growth rates [13]. This decreases women’s opportunity to participate in economic and educational activities [13].

Ethiopia is one of the low-income countries with the highest fertility and population growth rates and an unmet need for contraception [14]. According to the Ethiopian Demographic and Health Survey (EDHS), 2016, 22% of women of reproductive age had an unmet need for contraception [14]. However, other studies have suggested that women in the postpartum period have a higher unmet need for contraception, ranging from 37 to 47% [15, [16]. The gradual improvement in knowledge of contraceptive methods over time has positively influenced families to use family planning methods, though there are variations across countries, regions or communities [17]. Current evidence in LMICs indicated that fears of side effects and infrequent sex are the commonest reason for not using the contraceptive methods [18, 19]. Similarly, in Ethiopia, fear of side effects or health concerns and not resuming sexual intercourse and/or menstrual cycles are the commonest reasons cited by most women for not using contraceptive methods [20]. Nevertheless, these reasons could be prevented by providing continuous and quality family planning counselling. Evidence indicated that postpartum family planning counselling is associated with reduced unmet need for contraception [21]. A study from Nepal showed that women who received family planning counselling during either pre-discharge or post-discharge period were less likely to have the unmet need in the postpartum period than women who did not receive counselling [21]. Additionally, women who received family planning counselling in both the pre-and post-discharge period were less likely to have unmet needs than women who had not received counselling [21]. However, evidence is lacking in Ethiopia on the impact of family planning counselling during the maternal and child health services on the unmet need for contraception. This study examined the relationship between family planning counselling provided during the MCH services and the unmet need for contraception among women in the postpartum period in Ethiopia.

## Methods

### Study design and setting

We used a Performance Monitoring for Action (PMA) Ethiopian panel survey data, a national community-based cohort study conducted in five regional states - Tigray, Amhara, Oromia and South National, Nationalities and People (SNNPR), and Addis Ababa city. The survey used stratified multistage cluster sampling in the four regional states - Tigray, Amhara, Oromia and SNNPR - where each regional state was stratified into urban and rural strata. In contrast, the survey used a multistage cluster sampling technique without stratification in Afar and Addis Ababa. In the first stage, 265 *enumeration areas* (EA) created by the Ethiopia Central Statistical Agency were randomly selected as primary sampling units proportional to size. For the selected EA, household listings were developed by the survey team to serve as a sampling frame. In the second stage, a fixed number of 35 households per EA were randomly selected from the newly created household listings. Finally, all women within the selected households were screened for pregnancy status.

### Study population

**Fig. 1:**
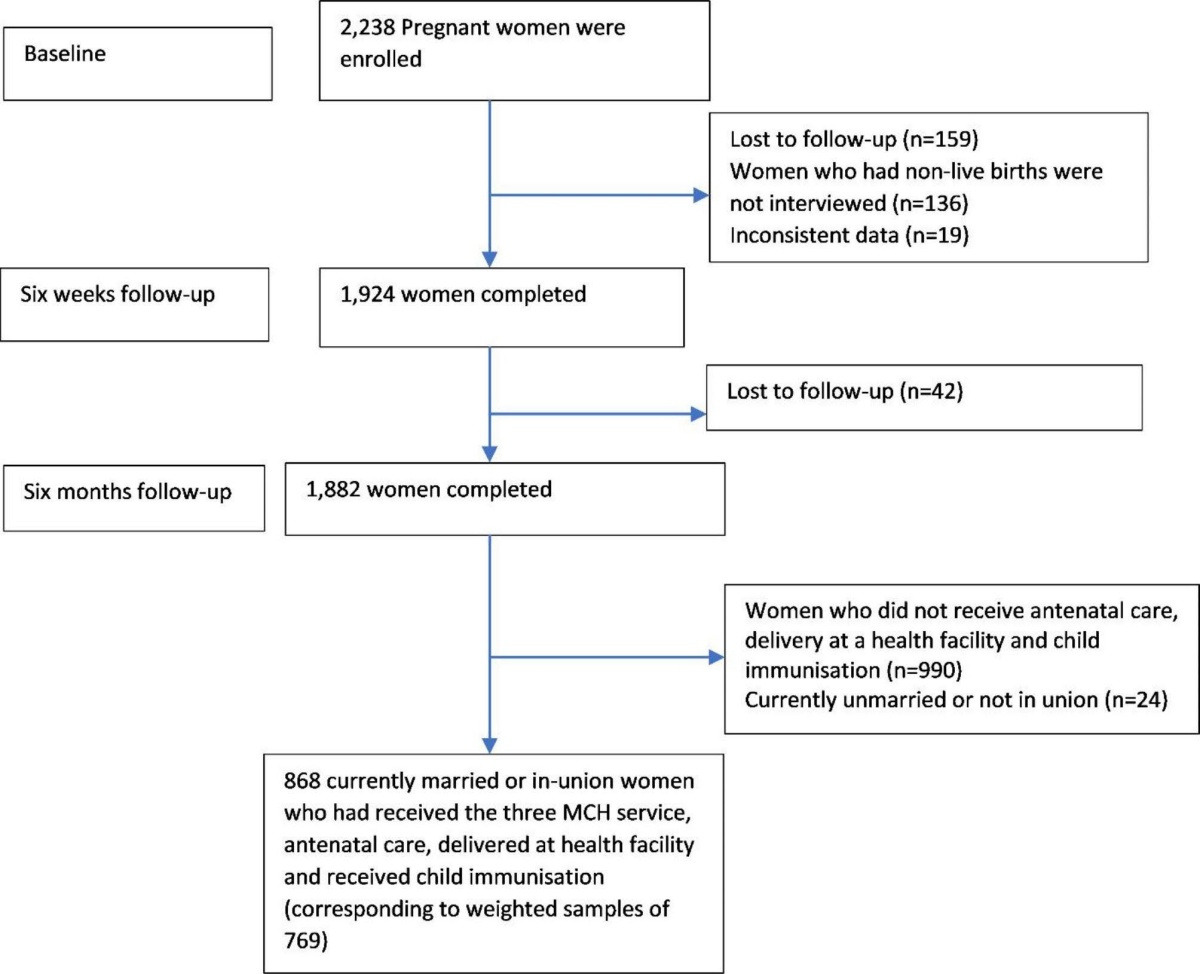
The sample selection process of the study

The study population was women in the postpartum period who had received the three MCH services. Of the 2,238 women enrolled at the baseline interview, 1882 completed the follow-up interviews. Exclusion of 990 women who did not receive the MCH service and 24 women who were unmarried or not in union resulted in an analytical sample of 868 women, corresponding to a weighted sample of 769 women in the postpartum period (Fig. 1).

### Data collection procedure

The baseline interview was conducted from September to December 2019 by trained resident enumerators (RE). The REs regularly monitored women’s deliveries and conducted follow-up interviews at approximately six weeks and six months postpartum between March and December 2020. In March, data collection was paused due to the COVID-19 restrictions in the country, but data collection resumed in July.

Women’s sociodemographic characteristics and family planning counselling during ANC were collected at the baseline interview. Family planning counselling during ANC (occurring after the baseline interview) and prior to discharge, pregnancy outcomes, and contraceptive uptake were collected at the six-week follow-up interview. Family planning counselling during child immunisation and modern contraceptive uptake during the first six months were captured at the six-month follow-up interview. All data were self-reported.

### Variables

#### Outcome variable

**Fig. 2:**
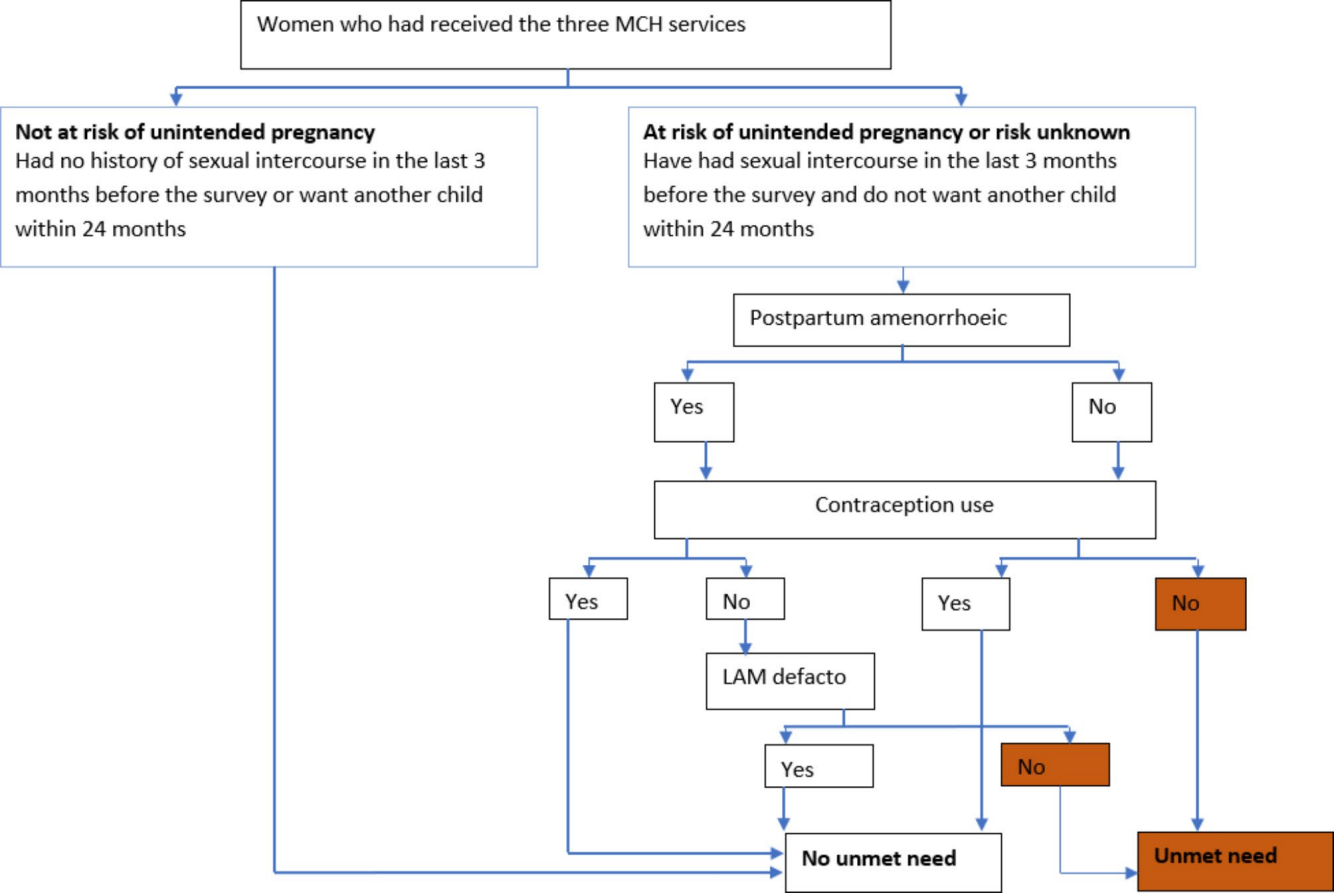
Definition of unmet need for contraception among women in the postpartum inEthiopia using the current status approach

An unmet need for contraception among postpartum women was the main outcome variable, as defined by the *current status approach* [6]. To determine the unmet need for contraception, we classified women as follows: If they are not postpartum amenorrheic and not using contraception, and have a risk of unintended pregnancy or unknown risk, or if they are postpartum amenorrheic, not using contraception, and have a risk of unintended pregnancy or unknown risk and not fulfilled the LAM defacto criteria (Fig. 2). We assessed the risk of unintended pregnancy based on women’s sexual history in the three months prior to the survey and their desire for another child within 24 months. Those who have had sexual intercourse in the past three months but do not want another child within 24 months were categorised as having a risk of unintended pregnancy, while those with unknown information about their sexual history and their next plan for another child within 24 months were classified as having an unknown risk. Additionally, we estimated exclusive breastfeeding using a 24-hour recall of the child’s feeding practices and considered women to fulfil the defacto LAM criteria if they were amenorrheic, 3–6 months postpartum following the recent birth, and exclusively breastfeeding [22].

#### Exposure variable

Family planning counselling was the primary exposure variable and was self-reported by the study participants. Counselling provided during ANC was captured both at baseline and by six-week follow-up interviews. Counselling provision status prior to discharge from the facility was captured by a six-week follow-up interview. Moreover, information on the provision of counselling during the child immunisation was captured at the time of the six-month follow-up interview as child immunisations are provided between the first six weeks and six months postpartum.

### Covariates

We controlled for various sociodemographic and childbearing characteristics, as well as health service utilization variables. Sociodemographic factors included household wealth status, women’s age, the highest level of education attained by the women, place of residence, and religion. Childbearing characteristics included parity, pregnancy intention of the most recent birth, and any experience of complications during the pregnancy and delivery. Additionally, health service utilization factors included the type of skilled health assistant during pregnancy and delivery, as well as the type of health facility where the delivery took place. A complete list and detailed definitions of the adjustment variables are found in the supplementary material (Additional file 1).

### Statistical analysis

We used a generalised linear regression model (GLM) fitted using a weighted modified Poisson distribution with a log link function to examine the association between family planning counselling provision during the MCH services and the unmet need for contraception among women in the postpartum period. We adjusted for maternal sociodemographic and childbearing characteristics and health service utilisation-related factors. All statistical analysis was carried out using Stata 16 [23].

## Results

A total of 769 women in the postpartum period who had received the three MCH services were included (Fig. 1). Approximately three-fifths (62%) of women were rural residents, and two-fifths of them had primary education of the highest level. Of the participants, 424 (55%) and 319 (42%) reported as they had experienced any complications during the recent pregnancy and delivery, respectively (Table 1). Of the participant women, 166 (22%) received family planning counselling during ANC visits, 212 (28%) prior to discharge from the facility and 215 (28%) during child immunisation. Women living in rural areas had received relatively more family planning counselling throughout the different MCH services (Table 1).

**Table 1:** Characteristics of study participants by family planning counselling provided at the different maternal and child health services (n = 769), Ethiopia, 2019–2020

Variable	Category	Total samples N = 769	Family planning counselling		
			During ANC N (%) 166 (22)	Prior to discharge N (%) 212 (28)	During child immunisation N (%) 215 (28)
Household wealth index	Lowest	172 (22)	48 (28)	46 (27)	49 (28)
	Middle	313 (41)	79 (25)	99 (32)	81 (26)
	Highest	283 (37)	39 (14)	67 (24)	85 (30)
Residence	Urban	293 (38)	48 (16)	76 (26)	80 (27)
	Rural	476 (62)	118 (25)	136 (29)	135 (28)
Living jurisdiction	Tigray	65 (8)	14 (22)	20 (30)	20 (31)
	Afar	1	0	0	0
	Amhara	192 (25)	44 (23)	37 (19)	29 (15)
	Oromia	299 (39)	42 (14)	80 (27)	89 (30)
	SNNPR	163 (21)	53 (32)	57 (35)	58 (36)
	Addis Ababa	49 (6)	13 (25)	18 (37)	18 (36)
Women’s age	< 25 years	275 (36)	49 (18)	74 (27)	80 (29)
	25–29 years	262 (34)	58 (22)	74 (28)	72 (27)
	30–34 years	130 (17)	32 (25)	33 (25)	36 (27)
	≥ 35 years	101 (13)	27 (27)	30 (30)	29 (29)
Women’s highest educational attainment	Not educated	218 (28)	51 (23)	54 (25)	45 (20)
	Primary	308 (40)	64 (21)	90 (29)	98 (32)
	Secondary	243 (32)	50 (22)	68 (29)	72 (30)
Religion	Orthodox	355 (46)	80 (22)	102 (29)	94 (26)
	Muslim	226 (29)	42 (19)	54 (24)	63 (28)
	Protestant	172 (22)	42 (24)	50 (29)	58 (34)
	Other	16 (2)	2	6	0
Parity	Nulliparity	247 (32)	34 (14)	48 (19)	80 (29)
	Primiparity	175 (23)	44 (25)	58 (33)	53 (30)
	Multiparity	245 (32)	64 (26)	77 (31)	63 (26)
	Grand multiparity	102 (13)	23 (23)	29 (28)	29 (28)
Pregnancy intention	Intended	545 (71)	121 (22)	149 (27)	148 (27)
	Unintended	224 (29)	45 (20)	63 (28)	67 (30)
Antenatal care provider	HP^1^	507 (66)	75 (15)	125 (25)	139 (27)
	HEWs^2^	55 (7)	21 (38)	24 (43)	21 (38)
	Both	207 (27)	70 (34)	63 (30)	55 (27)
Pregnancy complication	No	344 (45)	81 (24)	102 (30)	99 (29)
	Yes	424 (55)	84 (20)	110 (26)	115 (27)
Type of facility where given delivery	Health centre	440 (57)	112 (25)	118 (27)	125 (28)
	Hospital	295 (38)	48 (16)	85 (29)	80 (27)
	Other facilities	34 (4)	5	9	9
The type provider assisted the delivery	Nurse/Midwife/HEWs	348 (45)	82 (24)	103 (29)	97 (28)
	Skilled provider can’t distinguish	275 (35)	60 (22)	61 (22)	71 (26)
	Doctor	146 (19)	29 (20)	50 (35)	50 (34)
Complication during delivery	No	450 (58)	100 (22)	126 (28)	131 (29)
	Yes	319 (42)	65 (21)	86 (27)	84 (26)
Caesarean-section delivery	No	692 (90)	150 (22)	174 (25)	191 (28)
	Yes	77 (10)	15 (20)	38 (50)	24 (31)

In this study population, 234 (34%) women had an unmet need for contraception. The adjusted models showed that the risk of an unmet need for contraception was 12% less among women who had received family planning counselling prior to discharge than women who did not receive counselling (aRR 0.88; 95% CI 0.67, 1.16). Women who had received family planning counselling during child immunisation were 31% less at risk of having an unmet need for contraception than women who did not receive counselling during child immunisation (aRR 0.69; 95% CI 0.48, 0.98). However, women who had received family planning counselling during ANC had a 24% higher risk of an unmet need for contraception than women who did not receive counselling during ANC (aRR 1.24; 95% CI 0.93, 1.64) [Table 2]. However, the effect estimates for family planning counselling during ANC and prior to discharge were not precise (wide confidence interval).

**Table 2:** Association between family planning counselling during the maternal and child health services and unmet need for contraception among women in the postpartum, Ethiopia, 2019–2020

Timing of family planning counselling	Response	Unmet need for contraception		RR (95% CI)*	
		Yes	No	Unadjusted (n = 768)	Adjusted (n = 760)**
ANC	Yes	67 (41)	98 (59)	1.25 (0.95, 1.66)	1.24 (0.93, 1.64)
	No	195 (32)	408 (68)	1	1
Prior to discharge	Yes	69 (33)	143 (67)	0.94 (0.71, 1.25)	0.88 (0.67, 1.16)
	No	192 (35)	364 (67)	1	1
Child immunisation	Yes	56 (26)	159 (74)	0.70 (0.50, 0.98)	0.69 (0.48, 0.98)
	No	205 (37)	348 (63)	1	1

ANC-Antenatal care, ^*^RR – Relative Risk, CI- confidence interval

**Adjusted for household wealth status, residence, maternal age, maternal education, maternal religion, living jurisdiction (Afar was excluded), parity, pregnancy intention, type of provider who assist during recent pregnancy and delivery, type of facility where given birth, complication during pregnancy and delivery, and mode of delivery for the recent birth

## Discussion

This study determined the unmet need for contraception among postpartum women in Ethiopia and examined the association between receiving family planning counselling during the different MCH service contact points and the unmet need for contraception in the postpartum period. To our knowledge, this is the first study to examine the association between family planning counselling and the unmet need for contraception at the national level in Ethiopia. Our findings revealed that family planning counselling prior to discharge from the facility was associated with reducing the unmet need for contraception among women in the postpartum period. Moreover, family planning counselling during child immunisation was associated with reducing the unmet need for contraception. Similar to our study, a study from Nepal reported that family planning counselling provided pre- or post-discharge following delivery was associated with reduced unmet need for contraception in the postpartum period [21]. This is because family planning counselling services help dispel women’s misconceptions, increase women’s knowledge of contraceptive methods, help generate demand, facilitate informed decision-making, confer better contraceptive utilisation, and thereby lower the unmet need for contraception among those sexually active women who do not intend to get pregnant [24, 25]. Hence these findings highlighted the importance of strengthening the provision of integrated family planning counselling following childbirth across different contact points in the postpartum period.

Although this study found that family planning counselling during antenatal care (ANC) was associated with an increased risk of unmet contraceptive needs among postpartum women, the effect estimate was imprecise. This suggests that while family planning counselling during ANC may increase women’s demand for contraception, it may not necessarily result in the use of contraceptive methods. Sociocultural barriers such as religion and husband or family disapproval could hinder women from using contraceptive methods even after receiving counselling [14, 26]. Additionally, many postpartum women perceive a low risk of pregnancy during the first six months, which may prevent them from utilizing contraceptive methods even after counselling. Studies in Ethiopia have found that women in the postpartum period cite a low perception of pregnancy risk as a major reason for not taking up modern contraceptive methods [15, 27]. These findings suggest that family planning counselling should continue throughout maternal and child health services contact points to ensure that women use contraceptive methods as early as possible before the initiation of sexual intercourse following childbirth.

Ethiopia’s National Family Planning guideline recommends integrating family planning at all health service contact points [28, 29]. Nevertheless, this study revealed that only 22–28% of women received family planning counselling at all three contact points. The unmet need for contraception was 34%. Overall, poor awareness of the National Family Planning Guidelines by health care providers, care providers’ heavy caseloads, and inadequate resources, including materials for information, education and communication, likely contribute to low family planning counselling coverage [30–32]. Further research is recommended to explore why healthcare providers fail to provide integrated family planning counselling. Effectively using the opportunities of the maternal and child continuum of care to provide integrated family planning counselling and services would be an advantage in Ethiopia. The government, NGOs and other stakeholders may target continuous training for health care providers on integrated family planning counselling and regularly supervise, monitor, and evaluate health care workers’ performance. Moreover, healthcare workers also need to be compliant with offering integrated client-centred family planning counselling at all health service contact points dictated by government policy.

Unlike previous studies, we used the *current status* definition to estimate the unmet need for contraception, which helps understand the current risk of unintended pregnancy. This study used a cohort study design which allows the collection of exposure variables before outcome ascertainment. Additionally, this study’s analysis was based on national community-based representative data, making our findings nationally generalizable. Despite its strengths, this study also had several limitations. Family planning counselling was self-reported, which might result in recall bias. This study included family planning counselling received from healthcare workers during maternal and child health services. However, women might have received family planning counselling through other means such as media outlets or social media, or to some degree, they might have benefited from family planning counselling received before the recent pregnancy. In this study, approximately 16% of women were lost to follow-up. It is important to consider the potential impact of this loss on the results, as these women may have different fertility behaviours. The unmet need for contraception and associated family planning counselling may differ if these women were included in the analysis. Moreover, since this study was conducted among women who had access to a health facility, the findings may not be generalizable to all postpartum women. Women who did not visit a health facility for maternal and child health services could have different characteristics than those who did. Thus, the reported unmet need for contraception in this study may underestimate the true need, which could be higher among all postpartum women. Due to the unavailability of data, we did not assess supply-related factors, including the availability of contraception, healthcare workers’ knowledge and skills in counselling, family planning counselling quality, and women’s satisfaction with counselling services.

## Conclusions

Although a substantial proportion of women had missed the opportunities to receive family planning counselling during the MCH services, counselling during the postpartum periods, such as prior to discharge from the facility and particularly during child immunisation, was associated with reducing the unmet need for contraception among women in the postpartum period in Ethiopia. In contrast, family planning counselling during ANC was associated with increasing the risk of an unmet need for contraception. There was a high prevalence of an unmet need for contraception among women in the postpartum period. Our findings suggest the need for strengthening family planning counselling integration with MCH services in Ethiopia to ensure improved contraceptive uptake, reduced unmet need for contraception and the likelihood of unintended pregnancy and improved perinatal outcomes.

## Electronic supplementary material

Below is the link to the electronic supplementary material.

Supplementary Material 1

## Data Availability

The datasets supporting the conclusions of this article are available in public open access in the PMA repository (https://www.pmadata.org/data/request-access-datasets). Registration is required to get access. Competing interests. The authors declare that they have no competing interests.

## Footnotes

1 HP-Trained Healthcare workers.

2 HEWs-Health Extension Workers.
