# Supplementary material for: Achieving reductions in the unmet need for contraception with postpartum family planning counselling in Ethiopia, 2019–2020: a national longitudinal study
Source: Arch Public Health. 2023 May 1;81:79. doi: 10.1186/s13690-023-01096-1 (PMC10150151; doi:10.1186/s13690-023-01096-1)
Supplement: Supplementary file 1 — Supplementary Material 1 [file 13690_2023_1096_MOESM1_ESM.docx]

**Table A.1; Lists of adjustment variables and their definition**

| Covariate | Definition |
| --- | --- |
| Household wealth index | The household wealth index was calculated from the household's ownership assets materials used to construct the house, types of water access and sanitation facilities using the principal component analysis (PCA), considering the urban-rural differences. Later, the wealth index score was divided into three equal parts labelled as lowest, middle and highest |
| Residence (urban vs rural) | Participants' place of residence was coded as urban or rural. Urban was defined as a locality with 2,000 or more inhabitants. Moreover, all administrative capitals, including Region, Zone and Woreda, and localities in which urban dwellers' associations were established were considered urban residences, irrespective of the population size. Rural residences comprise all areas not classified as urban. |
| Living of jurisdiction | This study was conducted in five regional states and one city administration, including Tigray, Afar, Amhara, Oromia, SNNPR and Addis Ababa |
| Maternal age at enrolment | Maternal age at birth of the preceding child was categorised into <25 years, 25-29 years, 30-34 years and ≥35 years |
| Maternal education | Maternal educational status was categorised into not educated, primary, secondary, and higher (technical and vocational, and above) |
| Religion | Women's religion was classified into Orthodox, Muslim, Protestant, and others, including Catholic, Wakefetah, Traditional followers |
| Parity | As parity was captured during the baseline interview, it was considered excluding the recent birth and categorised as nulliparity (parity 0), primiparity (parity 1), multiparity (parity 2-4) and grand multiparity (parity ≥5) |
| Pregnancy intention | The pregnancy intention of the recent pregnancy was captured as 'then', 'later', and 'not at all'. We recategorised 'then' as intended pregnancy and 'later' and 'not at all' as unintended pregnancy. |
| Types of ANC providers | The providers who assisted women's ANC visits were categorised as health care providers, health extension workers (HEWs) or both health care providers and HEWs |
| Complications during the recent pregnancy | Women reporting any of the danger signs of pregnancy; *Severe headache with blurred vision, high blood pressure, oedema face/feet/body, convulsion/fits, vaginal bleeding before delivery, high fever, abnormal vaginal discharge (foul-smelling/dark), lower abdominal pain, worsening vision, particularly at night*, were considered to have experienced pregnancy complications |
| Type of health facilities where given birth | The types of health facilities where women delivered were categorised as government health centres, government hospitals, and others, including private and non-governmental health facilities |
| Birth assistant | The types of health care providers who assisted the recent delivery were grouped into Nurse/midwife, skilled attendants can't distinguish, Doctors and HEWs. |
| Complications during delivery | Women reported any of the following during delivery; *severe bleeding, Leaking/rupture of membrane and no labour pain for >24 hours, Leaking/rupture of membrane before nine months, malpresentation, prolonged labour (<12 hours), and convulsion/fits,* were considered to have experienced a complication during delivery. |
| Mode of delivery for the recent birth | Women were asked whether the recent birth was delivered by caesarean section or through vaginal |
